# Perspectives of current understanding and therapeutics of Diamond-Blackfan anemia

**DOI:** 10.1038/s41375-023-02082-w

**Published:** 2023-11-16

**Authors:** Yang Liu, Stefan Karlsson

**Affiliations:** 1 Center for Hematology and Regenerative Medicine, Department of Medicine Huddinge, Karolinska Institutet, Karolinska University Hospital, Stockholm, Sweden; 2 Division of Molecular Medicine and Gene Therapy, Lund Stem Cell Center, Lund University, Lund, Sweden

**Keywords:** Anaemia, Stem-cell research

## Abstract

Diamond-Blackfan anemia (DBA) is a rare congenital bone marrow failure disorder characterized by erythroid hypoplasia. It primarily affects infants and is often caused by heterozygous allelic variations in ribosomal protein (RP) genes. Recent studies also indicated that non-RP genes like *GATA1*, *TSR2*, are associated with DBA. *P53* activation, translational dysfunction, inflammation, imbalanced globin/heme synthesis, and autophagy dysregulation were shown to contribute to disrupted erythropoiesis and impaired red blood cell production. The main therapeutic option for DBA patients is corticosteroids. However, half of these patients become non-responsive to corticosteroid therapy over prolonged treatment and have to be given blood transfusions. Hematopoietic stem cell transplantation is currently the sole curative option, however, the treatment is limited by the availability of suitable donors and the potential for serious immunological complications. Recent advances in gene therapy using lentiviral vectors have shown promise in treating *RPS19*-deficient DBA by promoting normal hematopoiesis. With deepening insights into the molecular framework of DBA, emerging therapies like gene therapy hold promise for providing curative solutions and advancing comprehension of the underlying disease mechanisms.

## Introduction

Diamond-Blackfan anemia (DBA) is a congenital bone marrow (BM) failure disorder with erythroid hypoplasia that presents early in infancy (5–7 cases per million live birth) [1]. The disease is also categorized as ribosomopathy [2, 3]. Around 75% of cases of DBA are related to a heterozygous allelic variation in ribosomal protein genes (RP) of either the small or large ribosomal subunit [4]. Until now, more than 20 RP genes have been identified. In addition, mutations in non-RP genes such as *GATA1* and *TSR2* were also identified as a cause of the DBA phenotype [4]. Hematopoietic stem cell transplantation is currently the sole curative option for the treatment of DBA [1]. This treatment is, however, limited by the availability of suitable donors and the potential for serious immunological complications. A recent study demonstrated that gene therapy using a clinically applicable lentiviral vector could rescue the impaired anemia in both mouse and human *RPS19*-deficient DBA models, with a low risk of mutagenesis and a highly polyclonal insertion site pattern, providing evidence for a potential curable treatment for patients with *RPS19*-deficiency [5]. In the present review, we discuss recent molecular and genetic understanding and new advancements in novel therapeutics for DBA.

### History of the disease

**Fig. 1:**
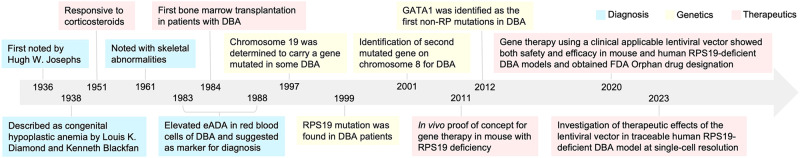
Timeline of understanding the history of DBA.

DBA was first reported by Hugh W. Josephs in 1936 [6], and more completely described by pediatricians Louis K. Diamond and Kenneth Blackfan who named the disorder as congenital hypoplastic anemia in 1938 [7] (Fig. 1). In 1951, corticosteroids were first reported to show therapeutic effects by Gasser [8], followed by a study of Diamond et al. indicating that a group of patients could respond to corticosteroid therapy [9]. In 1976, the first known bone marrow transplantation was performed on a 13-year-old boy with DBA who never responded to corticosteroid therapy and had received 238 transfusions, but iron chelation therapy showed no effects [10]. Initially, the treatment progressed well with erythroid precursors production was detected in the patient’s marrow for the first time in his life. However, the patient developed interstitial pneumonia and died 55 days after the transplant [10]. Elevated erythrocyte adenosine deaminase activity (eADA) in DBA patients was first reported and suggested as a marker for DBA by Diamond et al. in 1983 [11]. In 1997, a region on chromosome 19 was determined to carry a gene mutated in some DBA patients [12, 13]. Followed by this, mutations in the ribosomal protein S19 gene (RPS19) were found to be associated with disease in 42 of 172 DBA patients in 1999 [14]. Two years later, a second DBA gene was localized to a region of chromosome 8, and further genetic heterogeneity was inferred [15]. In 2012, the first non-RP gene, *GATA1*, was identified to have relationship with DBA, which broadened the understanding of molecular mechanism for DBA [16, 17]. The first in vivo prove-of-concept study by using gene therapy for the treatment of DBA was demonstrated in a mouse model with *rps19* deficiency in 2011 [18]. Followed by gradual optimization of the therapeutic vector, a clinically applicable lentiviral vector where the *RPS19* gene was driven by a cellular promoter, was shown to achieve both safety and efficacy in rescuing anemia and promote normal hematopoiesis in mouse and human *RPS19*-deficient models in 2021 [5]. Supported by this, the gene therapy strategy was approved for Orphan Drug Designation from FDA for further clinical trial investigation in patients with the *RPS19* mutation. Our recent study further demonstrated the therapeutic effects of the vector in a traceable precise *RPS19*-deficient human DBA model at single-cell resolution [19, 20].

### Clinical presentation

DBA is characterized by a paucity of erythroid progenitor and precursor cells in the bone marrow and red cell aplasia, and about half of the patients have physical malformations such as craniofacial defects, thumb deformities and short stature [4, 21]. Individuals with DBA also have a higher chance to develop cancer, including haematological malignancies (myelodysplastic syndrome, acute myeloid leukaemia) and solid tumors such as colon carcinoma and osteosarcomas [4, 21, 22].

Specifically, some DBA patients can also enter a state of remission [21, 23]. The DBA Registry defines “remission” as an adequate hemoglobin level without any treatment, lasting 6 months, independent of prior therapy [21]. The calculated likelihood of remission is 20% by age 25 years, with 72% experiencing a remission during the first decade of life [21]. Women also may relapse during pregnancy, with hormonal stress due to pregnancy appears to contribute to relapse [23, 24].

### Diagnosis and genetic screening of DBA

A detailed discussion about diagnosis was well described by Jeffrey M. Lipton et al. [23]. Briefly, the classic laboratory presentations of DBA include severe anemia (macrocytic or normocytic) and reticulocytopenia present within the first year of life, further supported by absence or limited cytopenias of other lineages, and a visible paucity of erythroid precursor cells in the bone marrow [23]. However, not all the patients present with the classic clinical criteria, and cases diagnosed in adults were also described [25]. In addition, bone marrow aspiration is also used to distinguish from other hypogenerative anemia and bone marrow failure. Apart from these, the eADA activity is a useful diagnostic biomarker for diagnosis [11, 21], which is elevated in 80% to 85% DBA patients [4, 26, 27] and it usually remains elevated even in patients who are in remission or are hematologically stable with corticosteroids treatment [21]. Ulirsch et al. also observed a significant association where *RPS19* and *RPS24* individuals appear less likely to have elevated eADA in a cohort study [4].

Molecular analysis is also used to identify genetic lesions. Genetic screening starts with targeted Sanger sequencing of *RPS19* (the most frequent genetic mutation) or directed next generation sequencing to analyse commonly mutated gene panels or all DBA related genes were applied according to the availability of the laboratory. Due to the limited incidence rate of the disease, DBA is not included in the universal prenatal screening for genetic disorders. However, when the DBA-causing pathogenic variant has been identified in an affected family member, it’s strongly advised to conduct prenatal testing for a pregnancy at increased risk and preimplantation genetic testing [21]. Details of DBA genetics will be discussed in the following paragraph.

### Genetics of DBA

#### RP genes

Around 70–80% of the DBA cases were found to have putatively causal haploinsufficient variants in genes encoding proteins that comprise the large 60S (RPL) or small 40S (RPS) ribosomal subunit, suggesting that these mutations mainly reduce ribosome levels, leading to a selective reduction in the translation of key genes involved in erythroid lineage commitment during hematopoiesis [4, 22]. Up to now, mutations in 23 RP genes have been identified and are heterozygous, which inherited in an autosomal dominant pattern (Table 1). Homozygosity is largely suspected to be lethal, supported by the lethality of homozygous RP gene mutations in several animal models [28, 29]. Among these, *RPS19*, *RPL5*, *RPS26*, and *RPL11* are the most frequently mutated RP genes [22]. A cohort study of 472 individuals with a clinical diagnosis of DBA showed that majority of the mutations are rare loss-of-function (LoF) alleles or missense, where 80% of mutations are a unique case [4]. Most putative causal mutations were typical LoF alleles or disrupted canonical mRNA splice sites, while the mutations predominately affect certain case of the extended consensus splice acceptor or donor site and a small number of rate mutations further from the exon-intron junction were also observed in the cohort [4]. Moreover, a mutation in the 3’UTR of *RPS26* was also reported, which was predicted to completely disrupt the polyadenylation signal by changing the consensus motif AA(T/U)AAA to AAGAAA [4]. There are also 7 candidates RP genes were considered to have relationship with DBA, which are extremely intolerant to LoF mutation [4].

**Table 1:** Clinical genetics of DBA and DBA-like syndromes.

	Gene	Transmission	Chromosome location	Percentage of patients
RPS	RPS7	AD	2p	<1%
	RPS10	AD	6p	3%
	RPS15A	AD	16p	<1%
	RPS17	AD	15q	1%
	RPS19	AD	19q	25%
	RPS20	AD	8q	<1%
	RPS24	AD	10q	2.40%
	RPS26	AD	12q	6.60%
	RPS27	AD	1q	<1%
	RPS28	AD	19p	<1%
	RPS29	AD	14q	<1%
RPL	RPL5	AD	1p	7%
	RPL8	AD	8q	<1%
	RPL9	AD	4p	<1%
	RPL11	AD	1p	5%
	RPL15	AD	3p	<1%
	RPL17	AD	18q	<1%
	RPL18	AD	19q	<1%
	RPL26	AD	17p	<1%
	RPL27	AD	17q	<1%
	RPL31	AD	12q	<1%
	RPL35	AD	3q	<1%
	RPL35A	AD	9q	3%
Non-RP genes	TSR2	X	X	<1%
	HEATR3	AR	16q	<1%
	GATA1	X	X	<1%
Candidate RP genes	RPS11	AD	19q	<1%
	RPL3	AD	22q	<1%
	RPL10	AD	X	<1%
	RPL10A	AD	6p	<1%
	RPL9	AD	17q	<1%
	RPL34	AD	4q	<1%
	RPL0	AD	12q	<1%

*AD* Autosomal recessive, *AR* Autosomal dominant, *X* X-linked.

There is no strong relationship with any specific mutation gene for the specific syndrome. However, neutropenia is more frequently associated with *RPL35a* [30–32], cleft palate and abnormal thumbs with *RPL5* and *RPL11* [33]. Specifically, patients with *RPL5* (83% on average) or *RPL11* (73% on average) mutations had higher chance with one or more congenital malformations, compared with mutations in the *RPS19* gene (34% on average) [4, 34]. Patients with *RPS24* (36%) and *RPL11* (29%) have higher chance to develop remission, compared with *RPS19* (8%) and *RPL5* (5%) mutations [4]. There is no significant difference in the treatment requirements for transfusion or corticosteroid dependence among mutations in the RP genes according to current experience [4].

#### Non-RP genes

In 2012, *GATA1* was identified as the first non-RP mutation in DBA using whole exome analysis [17]. *GATA1* is a hematopoietic master transcription factor that is both necessary for proper erythropoiesis and sufficient to reprogram alternative hematopoietic lineages to an erythroid fate [35]. The mutations were found at a splice donor site of the *GATA1* gene, and this leading to the impaired production of the full-length form of the protein, which required for normal erythropoiesis in humans [17, 36]. In addition, 2 RP chaperones, *TSR2* [37] and *HEATR3* [38], have also been identified in DBA patients. The ribosomal assembly factor *TSR2*, which is an *RPS26* chaperone (X-chromosomal gene encoding a direct binding partner of *RPS26*), has a critical role in ensuring adequate ribosome levels in hematopoietic progenitors [39]. Several individuals present with biallelic variants in *HEATR3* were shown to have association with DBA [38]. The *HEATR3* variants destabilize the protein, resulting in a reduction of nuclear *uL18* (*RPL5*) and impaired ribosome biogenesis independent of *p53* in CD34^+^ cells [38]. In particular, individuals with *HEATR3* variants exhibit more severe phenotype with bone marrow failure, short stature, facial and acromelic dysmorohic feature, and intellectual disability [38]. Specifically, *GATA1*-related and *TSR2*-related DBA are inherited in an X-linked manner, and *HEATR3* is inherited in a recessive manner [17, 38].

Moreover, *EPO* [40] and *CECR1* [41] were shown to be the DBA-associated genes. A homozygous recessive mutation in *EPO* (R150Q) was reported in an individual, and the mutation shows a mild reduction in affinity for its receptor but also altered binding kinetics, leading to less effective at stimulating erythroid cell proliferation and differentiation [40]. The cohort study identified recessive *CECR1* mutations in several individuals [41]. Each of the individuals presented with severe normocytic or microcytic anemia and bone marrow erythroid hypoplasia in infancy without any additional physical abnormalities. However, no abnormal rRNA maturation (typical in RP gene DBA) was observed in whole blood from 2 unrelated *CECR1* individuals. And these individuals were not observed to have elevated eADA [4]. Because of this, mutations in *CECR1* was regarded as DBA-like diseases, but screening for *CECR1* is highly recommended when individuals present with DBA [22].

### Molecular mechanisms of DBA

**Fig. 2:**
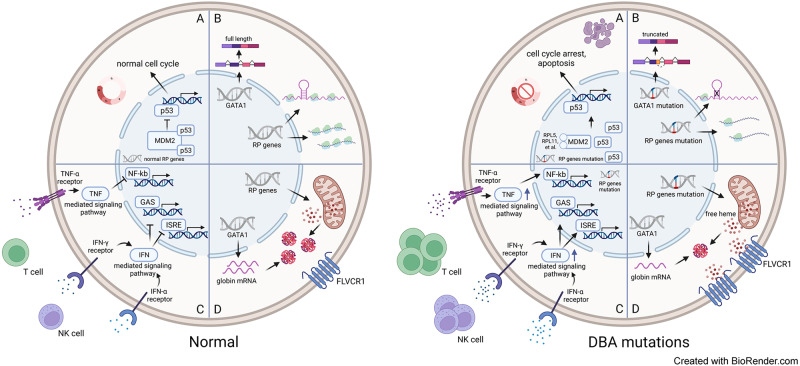
Summary of current understanding of molecular mechanisms for DBA. **A** *p53* activation and cell cycle arrest leading to ribosomal stress. **B** Translational dysfunction caused by *GATA1* and RP mutations. **C** Abnormal inflammatory signaling pathways due to RP mutations. **D** Unbalanced globin/heme synthesis caused by RP mutations.

The pathophysiology of DBA has not been fully understood. Since many mutations are RP genes, the mainly unsolved question is how the mutation in an RP gene leading to an aberrant ribosome assembly and impaired ribosomal biogenesis leads to the impaired erythroid defect [42]. Translation regulation, *p53* stabilization and cell cycle arrest, unbalanced globin/heme synthesis and autophagy were demonstrated to have relationship with DBA. Emerging evidence also indicated that inflammatory mechanisms may play a role in DBA (Fig. 2).

#### p53 activation and cell cycle arrest

Ribosomal stress was known to inhibit *p53* ubiquitination and induce *p53* transactivation, which leads to *p53*-dependent cell cycle arrest and apoptosis [43, 44]. Many RPs involved in the regulation of *p53* via interaction with its transcriptional target, *MDM2*, where RPs inhibit *MDM2*-medicated *p53* proteasomal degradation [45]. Several RP-mutations in DBA have been observed with activations of *p53* and target genes (especially *RPL5* and *RPL11*) in both animal models and patient samples [46–52]. By analysing the differentiation trajectories from megakaryocytic-erythroid progenitors (MEPs) to red blood cells and platelets, Lu et al. demonstrated that knockdown of *p53* leads to the reduction of MEPs and increase of erythroid progenitors [53, 54]. They also demonstrated that high cell cycle speed was required during MEPs fate decision, and erythroid progenitors have significantly more proliferation than megakaryocyte-committed progenitors by scRNA-seq analysis [54]. In addition, individuals with gain-of-function mutations in exon 10 of *p53* gene were reported to have DBA-like syndromes between DBA and dyskeratosis congenita [55]. *GATA1* was also demonstrated to have impact on *p53* inhibition [56]. All these findings indicated the essential role of RP-mutations in the induction of *p53* activation in the pathophysiology of DBA.

#### Translational dysfunction

Several studies of RP mutations have indicated at least modest reductions in overall protein synthesis [57]. It suggested that one main possibility is impaired translation of global or specific mRNAs in certain tissue leads to the specific ribosomopathy phenotype [36, 42, 58, 59]. The reduced RP expression was also known to lead to aberrant ribosome assembly and reduced ribosome levels. In most cases, the global protein synthesis is modestly reduced [42]. *GATA1* is the master hematopoietic transcription factor of megakaryopoiesis and erythropoiesis [60]. Mutation in the splice donor site of *GATA1* reduces the levels of full-length *GATA1* protein and can cause DBA in certain individuals [17, 36]. In addition, in patients with RP-mutation DBA, *GATA1* mRNA is poorly translated as the result of a highly structured 5’ untranslated region (5’UTR), and target genes of *GATA1* also showed globally and specifically reduction, which indicated the activity reduction of *GATA1* [36, 61]. It is still unknown how it impacts the reduction of *GATA1* mRNA translation, one possibility maybe the requirement of higher threshold for initiation of translation of *GATA1* mRNA compared to other genes [36, 62, 63].

#### Increased Inflammatory signaling pathway

Inflammatory signals are known to play a role for erythropoiesis. Overproduction of proinflammatory cytokines were shown to inhibit steady-state bone marrow erythropoiesis [64–67]. In contrast, inflammatory signals were demonstrated to induce stress erythropoiesis to maintain erythroid hemeostasis [68, 69]. Recent studies indicated inflammatory signatures would make impact on DBA, which may lead to the stress erythropoiesis. Elevated IFN-γ and TNF-α can be detected in DBA bone marrow plasma, and inflammatory signature was shown in erythroblasts and RBCs from DBA patients [70, 71]. By performing single cell RNA-seq (scRNA-seq) analysis using patient bone marrow HSPCs, increased IFN- α, IFN- γ, and TNF-α inflammatory pathways were identified in both RPS-DBA and RPL-DBA, with more obvious changes in RPS-DBA than RPL-DBA [70]. A previous reported zebrafish *RPL11* morpholinos also indicated increased inflammation [72]. Moreover, in patients responding to glucocorticoids treatment, increased type 1 interferon pathway was found to inhibit cell cycle progression by scRNA-seq analysis [73]. Interestingly, a low dose of interferon alpha treatment could promote RBC production in cells isolated from DBA [73]. Our recent study also identified enrichment of TNFα/NF-κB in gene edited human *RPS19*-deficient CD34^+^ cells by scRNA-seq analysis, while this was not observed in *RPS19*-deficient CD34^+^ cells treated with clinical applicable lentiviral vector [19]. The inflammatory signature also provides possible mechanism on how glucocorticoids exert their therapeutics in DBA [74, 75]. Taken together, both cell intrinsic and extrinsic defects may trigger inflammatory responses. Future studies about how inflammatory pathways contribute to the disease are worth to be explored.

#### Unbalanced globin/heme synthesis

The imbalance in excess free heme, which leads to production of reactive oxygen species were shown toxic to cells and leads to cell death and apoptosis [76, 77]. Similar to this, imbalanced globin and heme synthesis in primary DBA cells have been reported to lead to accumulation of free heme and heme toxicity in early erythroid precursors, which perturbs erythroid differentiation [78, 79]. In addition, mice with knockout of heme exporter, feline leukemia virus subgroup C receptor (*FLVCR1*), display impairment of erythropoiesis and congenital abnormalities as observed in DBA patients [80]. In some DBA patients negative for *RPS19* gene mutations, alternatively spliced isoforms of *FLVCR1* were also identified in immature bone marrow erythroid cells [81]. A recent study also found elevated *FLVCR1* expression in patients with *RPL11* and *RPL5* mutations, and decreased *GATA1* was also observed meanwhile [78]. Since α and β globins are transcriptionally regulated by *GATA1*, the reduction of *GATA1* was hypothesized leading to the imbalance heme/globin [82]. As *HSP70* is subjected to proteasomal degradation leading to decreased levels of *GATA1* in erythroid cells with *RPL5* and *RPL11* mutations [83], the author further demonstrated that overexpression of *HSP70* could protect *GATA1* and restore heme/globin balance. These findings imply the role of *FLVCR1* in the regulation of human erythroid cells through control of the heme content, which induce apoptosis on erythroid cells.

#### Autophagy

Autophagy is an important catabolic process that delivers cytoplasmic material to the lysosome for degradation. It promotes cell survival by elimination of damaged organelles and proteins aggregates, as well as by facilitating bioenergetic homeostasis [84]. A small molecular act through autophagy factor *ATG5* was identified to promote erythropoiesis and up-regulate expression of globin genes in induced pluripotent stem cells isolated from DBA patients and in vivo [85]. How the autophagy was regulated in DBA is not well understood. Autophagy was shown to affect erythropoiesis through degradation of the iron storage protein ferritin [86, 87]. And *Atg5*-deficient zebrafish are anemic, indicating that *ATG5* plays a role in erythroid development. More detailed studies are necessary to demonstrate the mechanism of autophagy on DBA.

### Treatments

#### Glucocorticoids, management and side effects

Glucocorticoids (GC) are the only widely used class of drugs in DBA since their first report in 1950s [8], about 80% patients respond to the therapeutic at the beginning, while half of these patients eventually discontinue GC treatment due to loss of response or severe side effects, such as growth retardation, pregnancy, etc [1, 21]. Until now, DBA is the only human disease in which steroids are administrated for years. Treatment with GC is not recommended in patients less than 1 year old due to growth inhibition [1, 88, 89]. The adequate response is defined as a hemoglobin level >90 g/l in combination with transfusion independency [1]. In general, treatment with GC is started with an initial dose of 2 mg/kg/day prednisone for a maximal period of 4 weeks [1]. In case of a response, slow tapering (below 1 mg/kg/day) is indicated to the lowest effective doses after initial 4 weeks [1, 21]. In most guidelines, 0.3–0.5 mg/kg/day of prednisone is regarded as the highest acceptable level to avoid long term toxicities [1, 22]. It is also recommended to take vitamin D supplementation to all DBA patients and perform periodic bone density measurements [1]. About 40% of case subjects remain dependent upon corticosteroids which increase the risk of heart disease, osteoporosis, and severe infections [1, 23]. For patients who make no or limited response on reticulocytes and hemoglobin levels, blood transfusion or hematopoietic stem cell transplantation are considered.

The mechanism of how GC works still not well understood and under investigation. A detailed review about the relationships of GCs on DBA through interacting with *GATA1*, *p53*, *c-myc*, *mTOR* and autophagy were well described by Zuzana Macečková et al. [90]. In untreated DBA patients, Wang et al. recently showed that erythroid progenitors entered S-phase of the cell cycle under considerable stress, leading to replication stress and activation of *p53* signaling [73]. However, in GC-responsive patients, cell cycle progression was inhibited by activation of the type 1 interferon pathway compared with GC-non-responsive patients [73]. Moreover, Iskander et al. also showed that the stress erythropoiesis in RPL-DBA exhibited disordered differentiation by an altered glucocorticoid molecular signature, including reduced *ZFP36L2* expression, leading to milder anemia and improved corticosteroid response compared with RPS-DBA [70]. In addition to this, Ryan et al. also demonstrated that dexamethasone treatment of peripheral blood progenitors can result in the expansion of a newly defined immature colony-forming unit (CD34^+^CD36^+^CD71^hi^CD105^med^) by activation of *p57*^*Kip2*^, which is a Cip/Kip cyclin-dependent kinase inhibitor. Notably, steroid resistance was shown to be associated with dysregulated *p57*^*Kip2*^ expression. In particular, this only happened in peripheral blood, not cord blood [91]. Taken together, a complex mechanism involving translation, proliferation and differentiation may all together contribute to the GC response.

#### Transfusion and management of iron overload

For patients who do not response to corticosteroid treatment have to be given blood transfusions [1, 21, 23]. Basically, patients require 10–15 ml/kg per RBC transfusion every 3–5 weeks to maintain hemoglobin levels above 80 g/l [1, 21, 23]. For infants and young children, higher levels of hemoglobin (>90 g/l) are required to maintain adequate growth and development [1]. However, the toxicity associated with iron overload, concomitant with chronic transfusion regimens, is a limiting factor for lifelong transfusion [1, 21]. The transfusion-associated iron overload is a leading cause of mortality in DBA patients in addition to HSCT-related mortality [1, 92]. Because of this, the effective and intense chelation therapy is necessary for DBA patients. Currently, the best and most feasible way to analyse iron overload is to perform magnetic resonance imaging (MRI)-based measurements of hepatic, cardiac and pancreatic iron burden [1]. If MRI is not available or applicable, serum ferritin levels of ≥1000 ug/l and/or transferrin saturation levels ≥75% are considered as a starting point for chelation therapy [1]. It is recommended to measure liver iron content every 12–18 months on chronic RBC transfusion treatment or screen for iron overload and start chelation therapy after 10–20 RBC transfusion (of 10–15 ml/kg), or when the MRI-measured liver iron concentration reaches ≥6–7 mg/g [1, 23]. Chelators such as deferoxamine or combination with deferasirox are used to achieve ferritin levels less than 500 ug/L and normal liver iron status by magnetic resonance imaging [22, 93, 94]. Specifically, deferoxamine is more preferred to use for infants as with supporting data [22].

#### Hematopoietic stem cell transplantation, managements and side effects

Currently, hematopoietic stem cell transplantation (HSCT) is the only curative treatment for DBA [1, 21, 22]. Standard indications for HSCT include resistance to GC treatment, chronic transfusion dependency and unacceptable GC toxicity [1, 95]. Recent studies suggest that HSCT should be recommended for transfusion-dependent children aged less than 10 years who make no response to GC or require high doses if a human leucocyte antigen (HLA) matched donor is available [1, 96–99]. The HLA-matched family donors are preferred donor type, and genetic screening of the affected gene for DBA to avoid an asymptomatic DBA carrier donor is necessary in cases with a known underlying genetic lesion. For patients with no mutation could be identified, it’s essential to assess potential related donors through erythrocyte adenosine deaminase analysis and a bone marrow test to exclude a silent carrier [98]. However, if this is not available, a 10/10 allele-matched unrelated donor is the best alternative [1]. For stem cell source, stem cells from bone marrow are more preferred than from peripheral blood due to lower risk of chronic graft versus host disease (GVHD) [96]. Umbilical cord blood derived stem cells from a sibling donor can also be considered if available, while transplantation with unrelated donors showed higher graft failure and transplant-related mortality rates [96]. For conditioning regimens, total body irradiation should be avoided in infants and not recommended for other DBA patients as it increases the risk of secondary malignancies as they already have higher risk for cancer predisposition. Myeloablative conditioning with busulfan, and more recently treosulfan, has been recommended as a means of favouring engraftment and reducing graft failure [96]. In recent years, low dose conditioning was suggested and has been demonstrated with efficacy in clinical trial [100, 101]. Our group also demonstrated the full correction of the hematopoietic phenotype in DBA mice given sublethal doses of irradiation, as well as in animals completely devoid of any proceeding irradiation [102]. In addition to this, antibody approach also showed promising effects with less toxic effects compared with conditioning [103, 104]. Donor rejection and GVHD also need to be considered when perform HSCT [1, 96]. Sufficient immunoablation (eg. Fludarabine) and serotherapy showed effects in reducing the risk of graft rejection and GVHD, especially for patients receiving unrelated donor [96, 98]. In addition, infertility is also a major concern after transplantation. Counselling about fertility preservation before transplantation and post-transplant follow-up are recommended [1, 96, 105].

#### Gene therapy, safety management and future perspectives

**Fig. 3:**
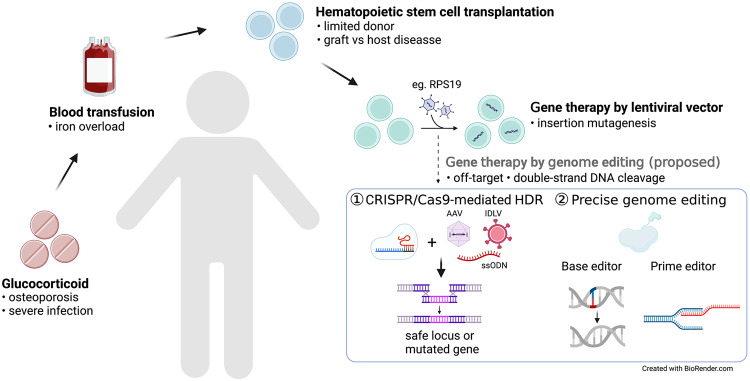
Summary of therapeutic alternatives for DBA.

Gene therapy using genetically engineered human hematopoietic stem and progenitor cells (HSPCs) is a potential therapeutic strategy for genetic blood disorders [106, 107] (Fig. 3). The use of self-inactivating lentiviral vectors for ex vivo gene correction of HSPCs has been successfully applied to treat primary immunodeficiencies [108, 109], haemoglobinopathies [110, 111] and metabolic disorders [112, 113] with superior engraftment and safer profile in patients [106, 107]. We recently demonstrated gene therapy using a clinically applicable lentiviral vector driven by a cellular promoter, EFS, could promote red blood cell production and normal hematopoiesis in a mouse DBA model with *RPS19* deficiency and human *RPS19*-deficient CD34^+^ HSPCs, with a low risk of mutagenesis and a highly polyclonal insertion site pattern [114]. Followed by this, similar strategies also showed rescue effects by other groups [115]. In addition, using lentiviral vector to express *GATA1* so that to promote red blood production is also being investigated, which also provides advantages for targeting most DBA mutations instead of a specific mutation [116]. However, the control of *GATA1* expression level is worth to be carefully investigated considering its regulation function as a transcription factor.

The successful development of gene therapy for *RPS19*-deficient DBA opens the possibilities for other mutations of DBA, such as *RPL5* and *RPL11*. In addition, gene therapy using CRISPR-Cas9 genome editing tools also showed therapeutic effects for genetic blood disorders such as sickle cell disease and beta-thalassemia [117–119]. The CRISPR-Cas9 derived editing tools such as high-fidelity (HiFi) Cas9, base editors and prime editors could improve editing efficiency with reduced off-targets or without double-strand DNA cleavage [119, 120]. These genome editing tools provide possibilities to directly edit mutated genes by using base editors [117], or edit erythroid-specific enhancer region of *BCL11A* with CRISPR-Cas9 [118], or deliver a full-length therapeutic gene site specifically using HiFi Cas9 and AAV via homologous recombination in HSPCs for hematologic disorders [119]. The above strategies can also be considered to develop gene therapy for DBA in the future. However, since *p53* activation was observed in patients with DBA, genotoxic risks are warranted to be considered when using gene editing [121, 122].

In addition to the ex vivo HSPC genetic manipulation, recent studies also demonstrated the possibility by using in vivo priming editing for the treatment of genetic blood disorder such as sickle cell disease in a mouse model [123], which provides significant advantages compared to the ex vivo gene therapy considering the needs of transplantation, ex vivo HSCs collection and myeloablative conditioning. This also opens the possibility for the development of in vivo gene therapy strategy for DBA. However, specific targeting to desired cell type such as HSPCs is essential to avoid off-targeting when performing in vivo delivery, which is also under investigation by using different delivery strategies such as viral vectors, lipid nanoparticles and virus-like particles [124].

## Conclusion

Followed by the first clinical report of DBA in the 1930s, a better understanding of the diagnosis, genetics, molecular mechanisms and novel therapeutics of DBA has been made through working together by patients, families, clinicians and researchers. With the recent advancement of next generation sequencing, more RP and non-RP genes were found to have relationship with DBA, which helps with clinical diagnosis and provides new clues to discover molecular mechanisms. The successful development of the mouse and human DBA models also provide support for the investigation of mechanisms and novel therapeutics. In terms of therapeutics, autologous gene corrected HSPCs using clinically applicable lentiviral vector in animal models showed curative treatment potential with both safety and efficacy, which also avoids challenges such as GVHD and donor limitation compared to HSCT. The rapid evolution of genome-editing and delivery technologies also provides opportunities to precisely correct mutations in DBA in the future. However, the molecular mechanism of DBA is still not fully understood, and novel therapeutics such as gene therapy should also be developed for other mutations of DBA. Future attempts in the investigation of these aspects will bring better understanding and more therapeutic alternatives for DBA.
