# Correction: Perspectives of current understanding and therapeutics of Diamond-Blackfan anemia

**DOI:** 10.1038/s41375-023-02101-w

**Published:** 2023-12-13

**Authors:** Yang Liu, Stefan Karlsson

**Affiliations:** 1 Center for Hematology and Regenerative Medicine, Department of Medicine Huddinge, Karolinska Institutet, Karolinska University Hospital, Stockholm, Sweden; 2 Division of Molecular Medicine and Gene Therapy, Lund Stem Cell Center, Lund University, Lund, Sweden

**Keywords:** Anaemia, Stem-cell research

Correction to: *Leukemia* 10.1038/s41375-023-02082-w, published online 16 November 2023

In this article the title was incorrectly given as Perspectives of current understanding and therapeutics of Diamond-Blackan anemia but should have been Perspectives of current understanding and therapeutics of Diamond-Blackfan anemia.

The original article has been corrected.

